# Chemically Protected Sodium Butyrate Improves Growth Performance and Early Development and Function of Small Intestine in Broilers as One Effective Substitute for Antibiotics

**DOI:** 10.3390/antibiotics11020132

**Published:** 2022-01-20

**Authors:** Huaibao Zhao, Hai Bai, Fuli Deng, Ruqing Zhong, Lei Liu, Liang Chen, Hongfu Zhang

**Affiliations:** 1 State Key Laboratory of Animal Nutrition, Institute of Animal Science, Chinese Academy of Agricultural Sciences, Beijing 100193, China; zhaohuaibao@caas.cn (H.Z.); 82101202366@caas.cn (F.D.); zhongruqing@caas.cn (R.Z.); liulei02@caas.cn (L.L.); 2 School of Life Science, Shanxi Datong University, Datong 037000, China; baihaiyx@126.com

**Keywords:** broiler, chemically protected sodium butyrate, antibiotic, growth performance, small intestine, morphology, ileal microbiota

## Abstract

The purpose of this study was to investigate the effects of chemically protected sodium butyrate (CSB) on growth performance and the early development and function of small intestine in broilers as one potential substitute for antibiotics. A total of 192 one-day-old Arbor Acres male broilers were randomly assigned into three dietary treatment groups (eight replicates per treatment): the control (CON) diet; ANT diet, CON diet supplemented with the antibiotics (enramycin, 8 mg/kg and aureomycin, 100 mg/kg); CSB diet, CON diet supplemented with 1000 mg/kg CSB, respectively. The results showed that dietary CSB and antibiotics addition significantly improved the growth performance of broilers by increasing the body weight gain (BWG) and feed conversion ratio (FCR) during different stages (*p* < 0.05). On day 21, the supplement of CSB in diet improved the structure of small intestine (duodenum, jejunum, and ileum) in broilers by increasing the ratio of villus height to crypt depth (VH/CD) (*p* < 0.05) and enhanced the butyric acid (BA) (*p* < 0.05) and total short chain fatty acids (SCFA) concentrations of small intestine (jejunum and ileum) compared with the CON and ANT diets. Besides that, the superoxide dismutase (SOD), total antioxidant capacity (TAC) and TAC to malondialdehyde (TAC/MDA) ratio of the ileal and jejunal mucosa were significantly higher (*p* < 0.05) in the CSB and ANT than in the CON. In addition, the supplement of CSB in diet markedly significantly enhanced α-amylase, lipase, and trypsin activities of the ileum (*p* < 0.05) as compared to the ANT diet. 16S rRNA gene sequencing indicated that CSB markedly increased the microbiota diversity of ileum in broilers at 21 days of age as compared to CON and ANT (*p* < 0.05). Furthermore, we found that *Firmicutes* was the predominant phyla and *Lactobacillus* was the major genus in the ileum of broilers. Compared with the ANT diet, the supplement of CSB in diet increased the relative abundance of some genera microbiota (e.g., *Candidatus_Arthromitus*, *Romboutsia*) by decreasing the relative abundance of *Lactobacillus*. Moreover, *Akkermansia* in the CSB was the highest in comparison to that in the CON and ANT. In addition, *Kitasatospora* that belongs to the phylum *Actinobacteriota* was only found in ileum of broilers fed the ANT diet. In summary, the supplement of 1000 mg/kg CSB in the diet improved the growth performance by promoting early development and function of the small intestine, which is associated with the regulation of intestinal flora and reestablishment of micro-ecological balance in broilers. Thus, CSB has great potential value as one of effective substitutes for in-feed antibiotics in the broiler industry.

## 1. Introduction

At present, broiler farming is characterized by large scale, high density, and exposure stress [1], which adversely affects physiological function of the gut, then decreases production performance [2,3]. Antibiotic growth promoters (AGP) as supplements in feed have been used to improve the growth and maintain the balance of intestinal ecosystem in poultry production for decades [4]. Unfortunately, this strategy has received under severe criticism due to the residue of antibiotics in environment, the enhancing resistance to antibiotics and even the dwindling efficacy in animals and humans [5,6,7], which has led to global actions to limit use of antibiotics in animal feeds. In 2006 the EU imposed a ban on the use of AGP in animal feeds [8]. From 1 July 2020, the prohibition of antibiotics in feeds has been fully implemented in China. However, the absence of AGP in feed leads to the high mortality and poor the growth rates of poultry, which greatly reduced production efficiency [9,10]. These exposing problems indicated it was necessary to search for alternative approaches in the absence of AGP supplementation. In poultry nutrition, many supplements have been proven to enhance feed efficiency and growth performance without the use of antibiotics [4,11,12,13]. Sodium butyrate (SB) is one of these supplements and is considered as a potential alter substitute for AGP because of its beneficial effects in poultry production [14].

The small intestine plays a major role in the digestion and absorption of nutrients. A healthy small intestine is essential for the optimal feed efficiency and better growth performance [15]. Sodium butyrate is easily converted to butyric acid (BA) in the digestive tract, where it improves the intestinal health by reducing the intestinal inflammatory response [16,17] and protecting the intestinal barrier function (including repairing the damaged intestinal mucosa [3,18], regulating the intestinal immunologic function [19,20] and stimulating the secretion of mucin [21,22] and antimicrobial peptides [23,24]); then, it increases the growth performance of poultry [3,25]. In contrast, some studies considered that SB addition had no effect on intestinal health and growth performance of poultry [17,26,27]. The variation in results may be due to the form of SB supplementation being different. For different forms of SB, for example unprotected or fat-coated, the gastrointestinal tract (GIT) segment wherein butyrate is released is diverse. Given the diversity of cell types, pH and microbiota composition in the different GIT segments of poultry, the release location may affect the observed responses to butyrate [28].

Good growth performance depends on a healthy small intestine that may be associated with the balance of intestinal microbiota [29]. Small intestinal microbiota plays an important role in affecting intestinal development and physiological functions, including the digestion and absorption of nutrients and the production of short chain fatty acids (SCFA) [30,31], although the populations of small intestinal microbiota (≈10^4^–10^8^ microbial cells) are very lower, compared with the large intestinal microbiota (≈10^10^–10^11^ cells/g) [32]. Lots of studies have shown that SB has the ability to balance the intestinal microbiota, but most of this work mainly focuses on the large intestine segment [17,19,26]. Few studies have evaluated the relationship between butyrate and the composition of small intestinal microbiota, which is more likely to respond to butyrate treatment and have a direct impact on the development and function of the small intestine.

Chemically protected sodium butyrate (CSB) is a special form of sodium butyrate that is protected by a physical and chemical matrix of buffer salts, which avoided dissociation at low pH in stomach or gizzard and is able to release enough butyrate in small intestine. In limited studies, Lan et al. [14] reported dietary inclusion of CSB increased growth performance of broilers, while Wu et al. [26] found that CSB could modulate the microbial community of the caeca, but there was no effect on the performance of broilers. The effects of CSB on small intestinal development, function and microbial community have not been deeply studied. It is well known that the early development and functional improvement of broiler intestine are very important for the whole growth period. Therefore, the purpose of the present study is to (1) investigate the influence of CSB as a potential alternative to antibiotics on growth performance in broilers at different stages, (2) determine the impact of CSB on small intestinal morphology and function including SCFA content, digestive enzyme activity and antioxidant capacity in 21-day-old broilers, and (3) examine the impact of CSB on the ileal microbial community of 21 day old broilers.

## 2. Results

### 2.1. Growth Performance

The effects of dietary CSB supplementation on the growth performance of broilers are presented in Table 1. As compared to the control (CON) diet, dietary CSB supplementation increased (*p* < 0.05) the body weight gain (BWG) significantly in the days 1–21 stage and tended to increase the BWG (0.05 < *p* < 0.1) in the days 1–42 stage, but there was no significant difference during the days 22–42 stage. Furthermore, CSB addition significantly (*p* < 0.05) decreased the ratio of feed intake to body weight gain (F/G) in days 1–21 and 1–42, indicating that CSB improved the feed conversion ratio (FCR), and tended to improve (0.05 < *p* < 0.1) in days 22–42. 

Compared with the CON diet, the BWG of broilers fed control diet supplemented with the antibiotics (ANT) diet increased significantly (*p* < 0.05) in days 22–42 and 1–42 stages, but there was no significant difference during days 1–21. The F/G ratio of broilers fed the ANT diet decreased significantly (*p* < 0.05) in days 1–21, 22–42, and 1–42 stages. In addition, there was no significant difference in the BWG and F/G ratio during each period between the CSB and ANT groups. Furthermore, there were no significant differences in feed intake among the three groups during the full period of this experiment.

### 2.2. Intestinal Histomorphologic Indices 

**Figure 1:**
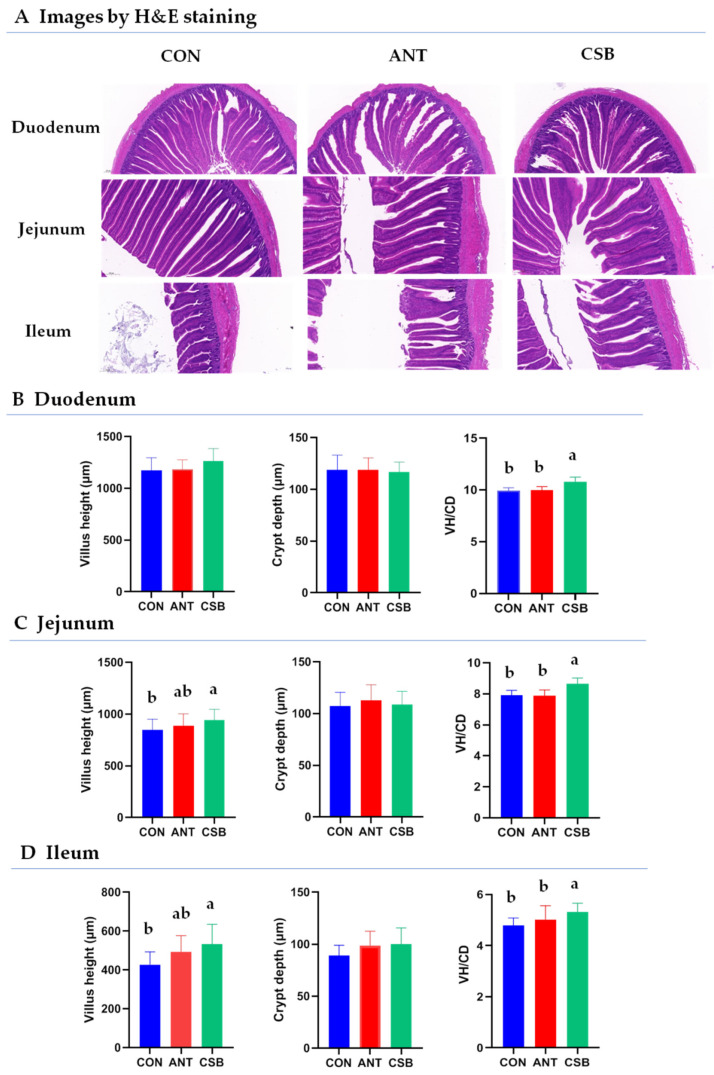
Effects of CSB on the morphology of the small intestine in broilers at 21 d of age. VH/CD: the ratio of villus height to crypt depth. Treatments: CON, the control diet; ANT, CON diet supplemented with antibiotics (enramycin, 8 mg/kg and aureomycin, 100 mg/kg); CSB, CON diet supplemented with 1000 mg/kg CSB; the same the following. Values are expressed as the mean ± SE, *n* = 8, the same the following. a,b: mean values were significant difference in the bars labeled with different letters (*p* < 0.05).

The morphology of the small intestine was determined by H&E staining. The villus height (VH), crypt depth (CD) and villus height/crypt depth (VH/CD) ratio of the small intestine in broilers at 21 d of age were measured (Figure 1). The VH/CD ratio in the duodenum, jejunum, and ileum for broilers fed the CSB diet was significantly higher (*p* < 0.05) than that of those fed CON and ANT diets. There was no significant difference in the duodenal, jejunal, and ileal VH/CD ratio between the CON and ANT groups. The VH in the jejunum and ileum in the CSB group was significantly higher (*p* < 0.05) than that in the CON group. There was no significant difference in duodenal VH among the three groups. In addition, the CD in the duodenum, jejunum, and ileum had no significant difference among the three treatments.

### 2.3. Activity of Small Intestine Digestive Enzyme 

**Figure 2:**
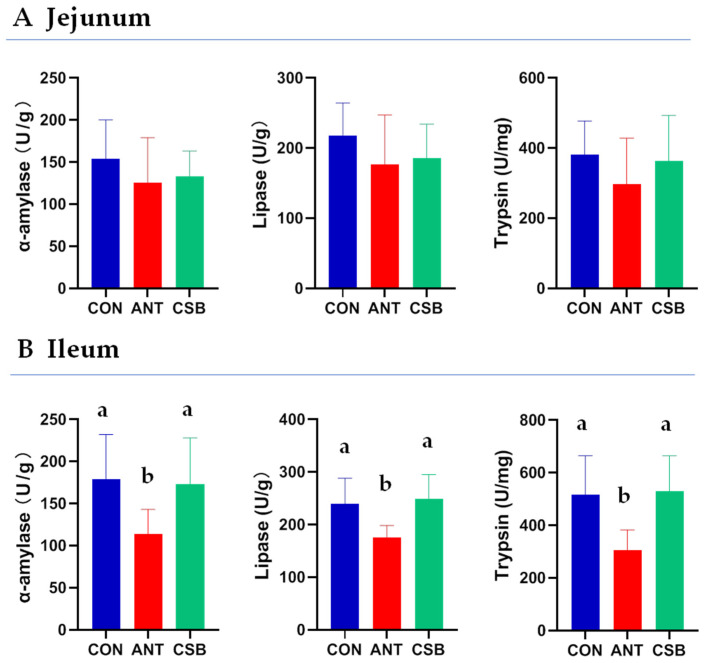
Effects of CSB on the intestinal digestive enzyme activities of broilers at 21 d of age. Activities of α-amylase and lipase were expressed as active unit per gram of chyme. Activity of trypsin was expressed as active unit per milligram of chyme. a,b: mean values were significant difference in the bars labeled with different letters (*p* < 0.05).

To examine the effect of CSB on the digestion of the small intestine, the intestinal digestive enzymes were determined in 21 d-old broilers (Figure 2). The activities of α-amylase, lipase, and trypsin in the jejunal chyme had no significant difference among the three treatments. Compared with the ANT diet, the activities of α-amylase, lipase, and trypsin in the ileal chyme of broilers fed CSB and CON diets were significantly increased (*p* < 0.05), while there was no obvious change between the CON and CSB groups.

### 2.4. Antioxidant Capacity of Intestinal Mucosa

**Figure 3:**
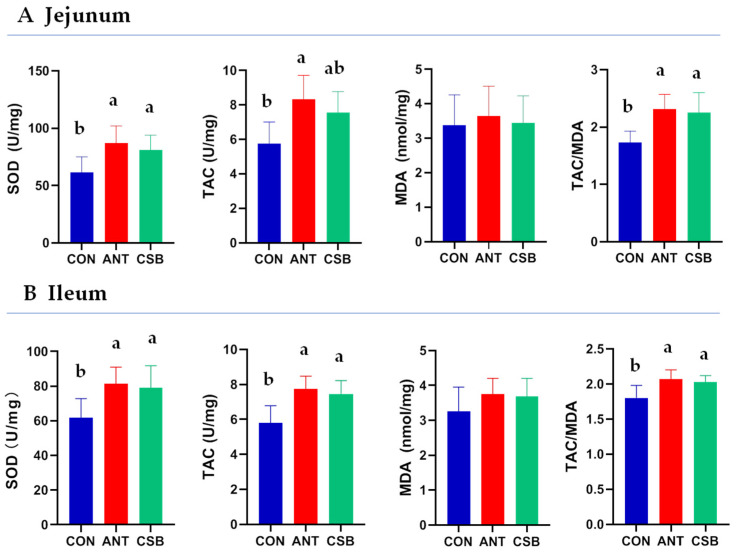
Effects of CSB on mucosal antioxidant capacity of the jejunum and ileum in broilers at 21 d of age. SOD: superoxide dismutase, U/mg prot.; TAC: total antioxidant capacity, U/mg prot.; MDA: malondialdehyde, nmol/mg prot.; TAC/MDA: the ratio of TAC to MDA. a,b: means that there was significant difference in the bars labeled with different letters (*p* < 0.05).

To examine whether CSB improves the antioxidant capacity of small intestine mucosa in broilers at 21 d of age, the superoxide dismutase (SOD), total antioxidant capacity (TAC), malondialdehyde (MDA) and the ratio of TAC to MDA (TAC/MDA) were determined (Figure 3). The SOD of the jejunual and ileal mucosa was significantly higher (*p* < 0.05) in the CSB and ANT than in the CON. Compared with the CON, the TAC of the jejunal mucosa significantly increased (*p* < 0.05) in the ANT and tended to increase (0.05 < *p* < 0.1) in the CSB, and the TAC of the ileum mucosa significantly increased (*p* < 0.05) in CSB and ANT. However, MDA were not significantly affected by CSB and antibiotics supplementation. Furthermore, the TAC/MDA (antioxidant balance) was significantly increased in the CSB and ANT compared with the CON.

### 2.5. SCFA Concentration in Intestine Contents

**Figure 4:**
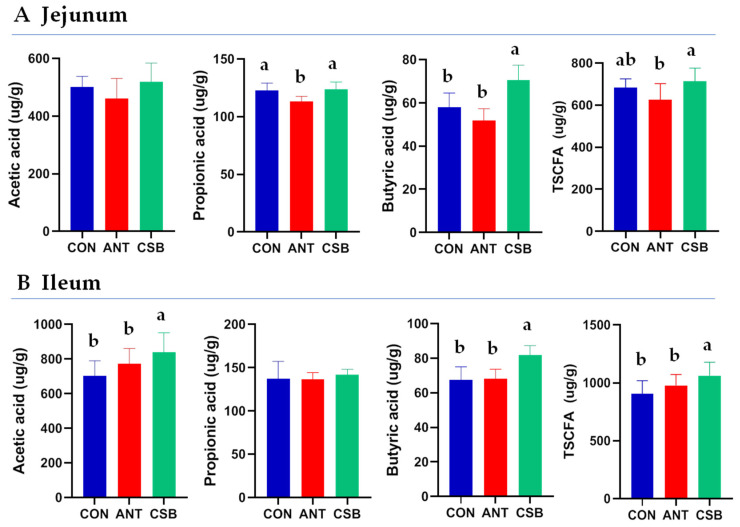
Effects of CSB on SCFA concentrations of the small intestinal chyme in broilers at 21 d of age. Acetic acid, propionic acid, butyric acid and total SCFA (TSCFA) concentrations were expressed as microgram per gram of chyme (μg/g). a,b: mean values were significant difference in the bars labeled with different letters (*p* < 0.05).

The effects of CSB addition on SCFA concentration of intestinal chyme in broilers at 21 d of age are presented in Figure 4. The concentration of BA in the jejunal chyme of broilers fed the CSB diet increased significantly (*p* < 0.05), while the concentration of propionic acid (PA) in the jejunal chyme of broilers fed ANT diet decreased significantly (*p* < 0.05) as compared to the CON diet. As compared to ANT diet, CSB addition increased significantly (*p* < 0.05) concentrations of PA, BA, and total SCFA (TSCFA) in the jejunal chyme. However, there was no significant difference in acetic acid (AA) concentration among the three groups. 

Concentrations of AA, BA, and TSCFA in the ileal chyme of broilers fed the CSB diet were markedly higher (*p* < 0.05) than those fed CON and ANT diets. However, concentrations of AA, BA, and TSCFA in the ileal chyme were not significantly different between the CON and ANT groups. In addition, there was no significant difference in PA concentration among the three groups. 

### 2.6. Microbiota Community Structure of Ileum

**Figure 5:**
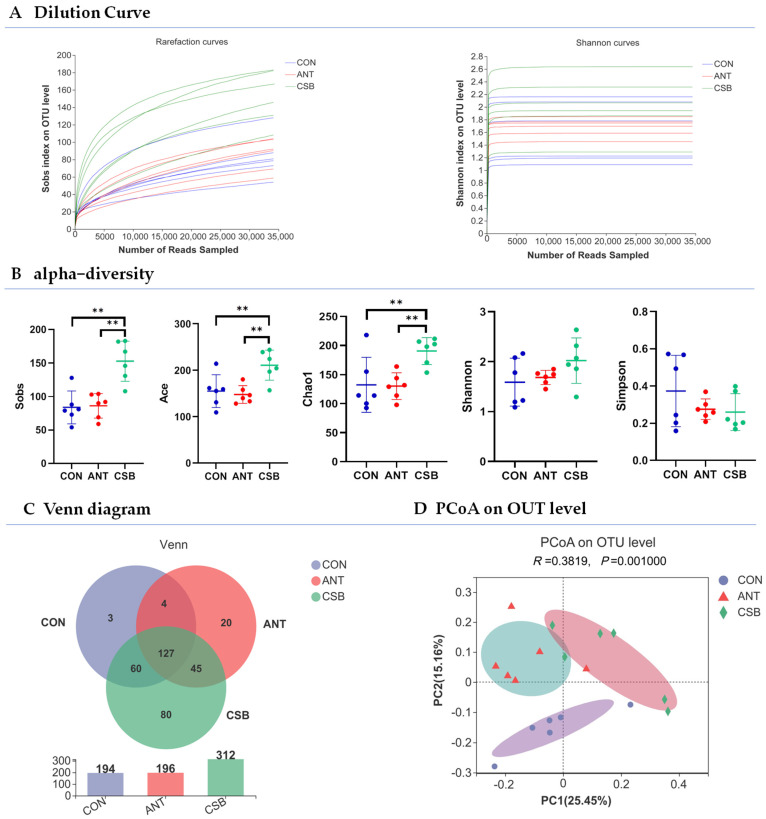
Effects of CSB addition on the microbial diversity of ileum in broilers at 21 d of age (*n* = 6). ** 0.001 < *p* ≤ 0.01.

Bacterial populations in ileal chyme of broilers at 21 d of age were measured using 16S rRNA gene sequencing to evaluate the impact of CSB on the microbiota of small intestine. A total of 814,514 quality sequences were obtained from all 18 samples in three groups, with a length of 390–430 bp, accounting for 95.5%. The end of the dilution curves tends to be flat (Figure 5A), indicating that the depth and number of sequences are credible. Beta–diversity was visualized using PCoA to elucidate the microbial composition. The two–dimensional plot of PCoA on the OTU level (Figure 5D) showed the microbial community from the three groups were divided into three clusters and occupied different positions, indicating that the microbial community structure is significantly different in the three groups. Sobs ACE, Chao 1, Shannon, and Simpson indices were used for evaluation of Alpha–diversity. The results shown compared with the CON and ANT diets, CSB addition increased significantly the sob, ACE and Chao indexes of broilers (*p* < 0.05), and had a certain impact on the Shannon and Simpson indexes (Figure 5B), revealing that CSB has the characteristics of improving the ileal microbial diversity of broilers. As shown in the Venn diagram (Figure 5C), 194, 196 and 312 OTUs were obtained from CON, ANT and CSB broilers, respectively, of which 127 OTUs were shared in three groups. Furthermore, there are 80 unique OTUs in the CSB, but only 3 and 20 unique OTUs in the CON and ANT, respectively. 

**Figure 6:**
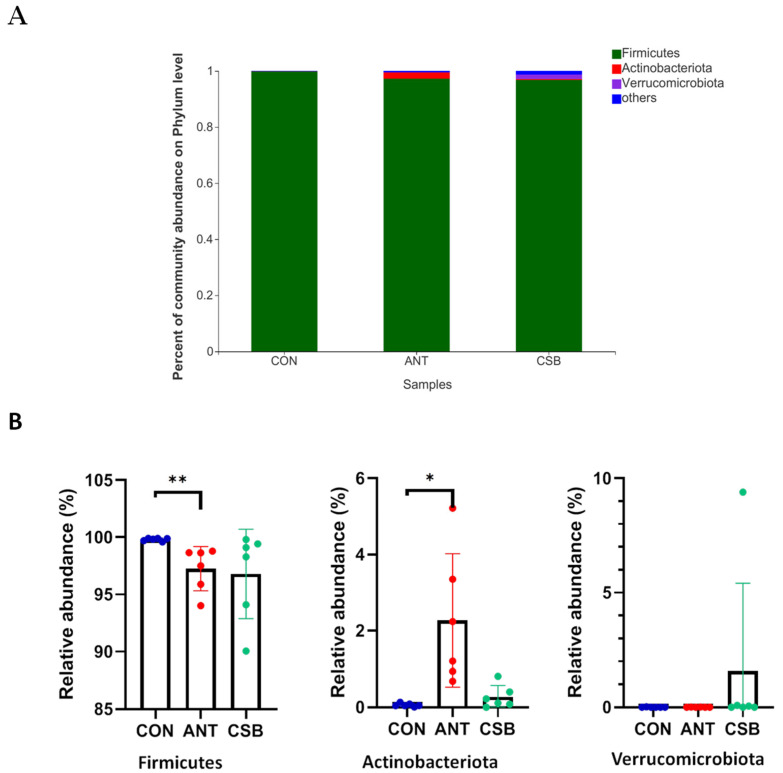
Effects of CSB on the ileal microbial community at the phylum level in broilers at 21 d of age (*n* = 6). (**A**) Percent of community on phylum level; (**B**) comparative analysis of relative abundance of major bacterial at phylum level. * 0.01 < *p* ≤ 0.05, ** 0.001 < *p* ≤ 0.01.

Furthermore, we analyzed the microbial community in the ileum chyme at the phylum and genus levels, as shown in Figure 6. *Firmicutes* is the predominant phylum of ileum, accounting for more than 96% of the total sequences (Figure 6A). Furthermore, the main bacterial phyla among three groups were compared (Figure 6B). Compared with the ANT (97.25%) and CSB (96.80%), the relative abundance of *Firmicutes* was the highest (99.81%) in the CON (*p* < 0.05). However, the relative abundances of *Verrucomicrobiota* and other phyla in the CSB were the highest compared with the CON and ANT. In addition, the relative abundance of *Actinobacteriota* significantly increased in ANT (*p* < 0.05), from 0.06% in the CON and 0.32% in the CSB to 2.27% in the ANT. 

**Figure 7:**
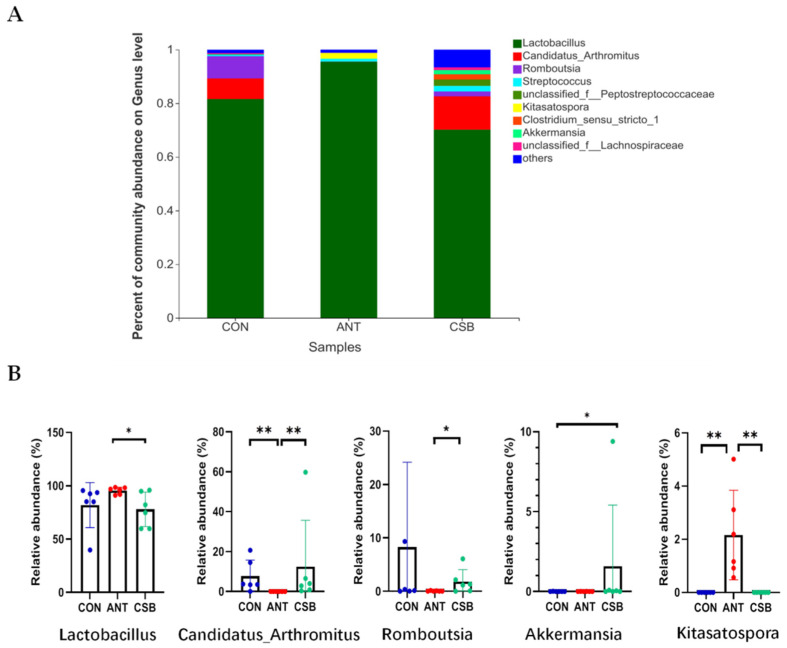
Effects of CSB on the ileal microbial community at the genus level in broilers at 21 d of age (*n* = 6). (**A**) Percent of community on genus level. (**B**) Comparative analysis of relative abundance of major bacterial at genus level. * 0.01 < *p* ≤ 0.05, ** 0.001 < *p* ≤ 0.01.

*Lactobacillus* is the predominant genus of ileum, accounting for more than 70% of the total sequences (Figure 7A). Furthermore, the relative abundance of *Lactobacillus* (95.51%) was higher in the ANT than that in the CON (81.58%, *p* = 0.065) and CSB (70.18%, *p* < 0.05) (Figure 7B). Furthermore, the relative abundance of *Kitasatospora* (2.16%) in the ANT significantly increased (*p* < 0.05) as compared to the CON (0.000%) and CSB (0.001%). However, the relative abundance of *Candidatus_Arthromitus* in the ANT was the lowest (0.002%) as compared to the CON (8.08%) and CSB (12.43%, *p* < 0.05). Besides that, the relative abundance of *Romboutsia* (0.043%) was lower in ANT than that in the CON (8.29%, *p* = 0.090) and CSB (1.81%, *p* < 0.05). In addition, the relative abundance of *Akkermansia* in the CSB was the highest (1.59%) in comparison to that in the CON (0.0039%, *p* < 0.05) and ANT (0.0049%, *p* = 0.089).

## 3. Discussion

For decades, the use of AGP at a sub therapeutic level in feed has shown a positive effect on the intestine health and production performance of broilers [33]. This study also proved that oral administration of AGP (Aureomycin and Enramycin) was able to enhance the growth performance of broilers. As potential alternatives for AGPs in poultry production [5], SB and its coated forms have a positive effect on broiler performance without the use of antibiotics [19,25,34,35,36]. In the current study, we have also demonstrated dietary 1000 mg/g CSB (SB protected by a double buffer system) supplementation could improve the growth performance of broilers as shown by increasing the BWG and decreasing the F/G ratio, as well as the enhancement of early development and function of the small intestine such as improving intestinal morphology, digestive enzymes activities, antioxidative capacity, and SCFAs levels, which may be related to the changes of ileum microbiota. 

The current study proved that CSB addition improved BWG and feed conversion ratio (FCR) of broilers in days 1–21 and 1–42; however, there was no significant difference during the days 22–42. Similarly, Hu and Guo [37] reported that the BWG of broilers during days 0–21 increased linearly as the dietary supplementation of SB increased, but there was no significant difference in days 22–42 and 1–42. However, Sikandar et al. [38] found that fat-coated SB improved BWG and FCR of broilers during days 22–35, but had no effect during days 0–21. On the contrary, some studies indicated that SB and its coated forms or BA had no favorable effects on performance [17,26,39]. Evenly, fat-coated SB supplementation decreased significantly BWG and FI of broilers in days 0–21 [22]. These inconsistent results may be associated with differences in the age and health status of broilers, feed nutrition and composition, and the form and concentration of butyrate used. This is a fact that fat-coated SB is not released completely, as the intestinal function and digestive enzyme activity of day old chicks are not fully developed [5], which causes lowered BWG and poor FCR in the starter period, then may impact performance in the overall period. Moreover, broilers raised in the environment with less pathogens can reduce the beneficial effect of AGPs and alternatives on growth performance [12,27]. 

Growth and production of poultry mainly depend on the digestion and absorption of dietary nutrients by the small intestine [15]. The pancreas can secrete a series of digestive enzymes into the small intestine, which are very pivotal for the digestive efficiency of feed. BA has been shown to stimulate the pancreatic exocrine, thereby increasing the activity of digestive enzymes such as amylase in the intestine [40]. Wu et al. [41] found a significant elevation in the levels of the pancreatic α-amylase and lipase activities of chicken fed on SB-enriched diets. Furthermore, Jazi et al. [42] reported dietary supplementation with butyrate salt ameliorated the negative impact of the challenge with *Salmonella Typhimurium* on the amylase and protease activities in the jejunum of broiler chickens. However, this study showed that dietary CSB supplementation did not improve the intestinal digestive enzyme (α-amylase, lipase, and protease) activities of broilers as compared to the CON diet. This may be related to the health status of broiler intestine itself in the trial, but it needs to be further studied. Interestingly, we observed a significant decrease in the α-amylase, lipase, and protease activities of ileum in broilers fed on ANT diet, indicating that antibiotics reduced the digestibility and utilization of feed. This indirectly showed that the improvement of growth performance of broilers by antibiotics was not related to the enhancement of feed digestibility.

Intestine for the optimal absorption is characterized by a large surface area covered with healthy and long villi with shallow crypts [5]. VH, CD and VH/CD are important indicators to evaluate the function of the small intestine [43]. In principle, the higher VH and VH/CD and the lower CD mean a better the intestinal structure, and a stronger digestion and absorption capacity of nutrients [44,45]. The current study showed that CSB supplementation significantly increased the VH and VH/CD of small intestine of 21 d-old broilers, indicating the ability of CSB to improve the early development of the small intestinal villi and absorption function. Similarly, lots of studies have demonstrated the beneficial effects of SB and its coating forms on VH, and/or CD and/or VH/CD at different phases of broiler growth, although these results had some variations [22,26,34,38,46]. For example, Chamba et al. [34] reported partially fat-coated SB addition increased the VH of jejunum and ileum in broilers, while González-Ortiz et al. [22] found SB increased the VH/CD of ileum in broilers, mainly due to the numerically lower CD. The discrepancy in results may be related to the fact that the gastrointestinal tract (GIT) segment wherein SB and its protected forms are released is different. In poultry, SB is easily absorbed in the upper GIT (e.g., crop, stomach), while fat-coated SB allows butyrate to reach the distal end of GIT (e.g., ileal, cecum) [28]. Earlier releasing of SB in the foregut (jejunum, ileum) can improve the development of villi and enhance the digestibility of nutrients, but later releasing time in the cecum can inhibit pathogenic bacteria [15]. The current study showed the CSB addition could significantly increase the concentration of BA in the small intestine, indicating the release characteristics of CSB in the small intestine. This may be the reason why CSB promotes small intestine villi development. 

Oxidative stress (OS), considered to be a state of imbalance between antioxidation and oxidation, can seriously debase the productivity and even lead to animal death [47]. Investigations have shown that SB can improve the antioxidant capacity and protect against OS damage on animals [14,48,49]. The intestine is highly susceptible to OS, which causes the waste of nutrients as well as gut dysfunction and body disorders. Micro-encapsulated SB addition could attenuate OS response induced by corticosterone exposure through increasing catalase activity and decreasing the MDA level of intestinal mucosa in broilers [50]. Similarly, this study showed that the addition of CSB in diet enhanced the intestinal antioxidant capacity by increasing the SOD and T-AOC activities in the jejunal and ileal mucosa of broiler at 21 d. TAC/MDA represents the antioxidant/oxidative balance, is used as an index of OS status [51,52]. Furthermore, we found that CSB improved the small intestinal OS status through the increase in TAC/MDA ratio (antioxidant balance) which was caused by the increase in TCA. 

The current study showed the dietary CSB addition increased markedly the concentrations of SCFA (AA, PA, BA) of the jejunum and ileum to different extents in broilers at 21 d, which is similar to the previous report [26]. The increase in SCFA contributed to the reduction in intestinal pH that affected beneficially the host. González-Ortiz et al. [22] showed that dietary fat-coated SB supplementation could increase the concentration of BA for the ileum chyme in broilers at 21 d. On the contrary, Hu and Guo [37] found that SB had no effect on concentrations of SCFA. These different results may be related to the fact that the release and utilization of CSB is located in the foregut, whereas the absorption of unprotected SB was in the upper gastrointestinal tract and the release of fat-coated SB was in the hind-gut [15]. SCFA are metabolite products of carbohydrate fermentation by gut microorganisms [53]. The intestinal SCFA levels were closely related to the number and composition of intestinal microbiota. This study showed CSB increased the diversity and composition of the ileal microbiota of broilers at 21 d significantly, which contributed to the understanding of the CSB-enhanced the SCFA levels in the small intestine, at least in part due to alterations in the ileal microbiota of broilers. Generally, *Firmicutes* and *Bacteroidetes* are considered to be the main microorganisms producing SCFA [54]. However, we found that the ileal *Firmicutes* was lowest through CSB treatment, and *Bacteroidetes* were rare in all samples. These findings suggested the increase in ileal SCFA in broilers fed the CSB-enriched diet may be caused by the fermentation of other microbiota, such as *Ruminococcaceae*, *Akkermansia*, which were different from the relationship between the abundance of *Firmicutes* and *Bacteroidetes* and SCFA levels in the traditional sense [55]. 

A healthy gut is closely related to the balance of intestinal flora. In other words, intestinal microbiota disorder can lead to poor intestinal health, such as slowing the rate of intestinal epithelial renewal and increasing the susceptibility to pathogen colonization, and then reducing production efficiency of poultry [56]. It is well known that SB can improve intestinal health by inhibiting the number of pathogenic bacteria (e.g., *salmonella*) and/or increasing the number of beneficial bacteria (e.g., *lactobacilli*) of gut in poultry [15,57]. Moreover, lots of studies have shown that SB supplementation can affect the intestinal cecal microbiota of broilers [17,19,26]. Limited studies explored the relationship between butyrate and small intestinal microbiota. Yang et al. [58] found that the addition of 3000 ppm butyrate in the form of butyrate glycerides in diet did not affect the alpha-diversity of ileal microbiota but altered its composition in broilers. However, we found that CSB addition significantly enhanced the microbiota diversity of ileum in broilers based on bacterial 16S rRNA gene sequencing analysis, hinting that CSB has a powerful capacity to intervene with the microbial community. Furthermore, antibiotics lowered significantly the a-diversity of the ileal microbiota as compared to CSB, which might be due to broad-spectrum anti-bacterial activity of antibiotics. High intestinal microbiota diversity generally contributes to general health and the growth performance of animals [59], which is conducive to an understanding of CSB improving the small intestine’s early development and function in broilers, at least in part attributed to high diversity of the ileal microbiota. 

Generally, *Firmicutes* and *Bacteroidetes* were the dominant phyla in the cecum of broiler chickens [17,26,60]. However, we found *Firmicutes* was the major phylum and yet *Bacteroidetes* was rare in the ileum of broilers, which is similar to the observations of Xiao et al. [56]. This might be related to the difference of location structure and function of cecum and ileum, and simultaneously different gut microbes contributing to different gut functions [56]. *Firmicutes* plays an important role in protecting intestinal barrier function and is beneficial to intestinal health [61]. Furthermore, we found *Lactobacillus* is the dominant genus in this phylum which is consistent with previous studies [56,62]. *Lactobacillus* is a recognized beneficial bacterium phylum, which can provide nutrition for the intestine and prevent the colonization and growth of pathogenic bacteria [62].

This study showed that antibiotic (aureomycin and Enramycin) addition increased the relative abundance of ileal *Lactobacillusm*. Similarly, Huang et al. [62] reported antibiotic (chlortetracycline) increased *Lactobacillus* of the foregut in AA broilers. In addition, *Kitasatospora* that belongs to the phylum *Actinobacteriota* was only found in ileum of broilers fed the ANT diet. These results indicated that the inhibitory ability of antibiotics to different bacteria genera is different. *Actinobacteriota* can produce lots of useful chemicals and metabolites, including a variety of antibiotics. Thus far, at least 50 bioactive compounds have been found from *Kitasatospora* [63]. It is speculated that the increase in the production of natural antibiotics in the gut will amplify the antibacterial effect of antibiotics applied, thus benefiting the host [62]. Therefore, we considered that antibiotics (aureomycin and Enramycin) increased growth performance of broilers by improved intestinal health, at least in part attributed to high levels of *Lactobacillus* and *Kitasatospora* in ileum.

Interestingly, we found that CSB addition enhanced the relative abundance of *Verrucomicrobia* phylum and *Akkermansia* genus in broiler ileum. *Akkermansia* is the only genus of the *Verrucomicrobia* phylum found in gastrointestine [64]. *Akkermansia* is associated with intestinal health and has been found to improve intestinal barrier function [65]. It has been proved that SB improved the intestinal health involved in protecting the intestinal barrier function [3,66]. Therefore, it seems reasonable to speculate that dietary CSB addition enhance intestinal development and function of broilers, which is closely related to the increase in *Akkermansia*, then the improvement of intestinal barrier function. On the other hand, *Akkermansia* can degrade mucin and competitively inhibit the growth of other pathogenic bacteria which degrade the mucin [67], which is considered to be a promising probiotic [43]. Degradation properties of *Akkermansia* lead to the production of SCFAs [54,68], and previous studies have also shown that SCFA can stimulate the expression and secretion of mucin [69,70]. These results reveal a subtle interrelationship: *Akkermansia* degrades mucin to produce SCFA, while SCFA simultaneously stimulates goblet cells to secrete more mucin. Here, we agree with the hypothesis of the *Akkermansia*–SCFA–mucin balance which is stable within a certain range [54]. However, this needs to be further researched.

## 4. Materials and Methods

### 4.1. Animals and Experimental Design

A total of 192 one-day-old Arbor Acres (AA) male broilers (Beijing Huadu Broiler Company, Beijing, China) were used in the trial. All broilers were weighed and randomly divided into three dietary treatment groups according to the principle of similar weight, with 8 replicates in each group and 8 chicks in each replicate. The growth period lasted 42 days. Three dietary treatments were the control (CON) diet, the basal corn–soybean meal diet; ANT diet, CON diet supplemented with the antibiotics (Enramycin, 8 mg/kg and Aureomycin, 100 mg/kg); CSB diet, CON diet supplemented with 1000 mg/kg chemically protected sodium butyrate (CSB), respectively. The composition and nutrient levels of the basic corn soybean meal diet are shown in Table 2, following the broiler feeding standard (China, 2004). Antibiotics and coccidiostats were not used in the above basic meal diet. The supplementation CSB (Provided by Beijing Shengtaiyuan Bio-Technology Co., Ltd., Beijing, China) contains 54% sodium butyrate, which is protected by a physical and chemical matrix of buffer salts, avoiding it dissociation by stomach pH. During the trial, chicks were raised in wire cages with the plastic feeder and drinking trough and provided with mash feed and water ad libitum. The temperature, humidity and ventilation of the chicken house is controlled according to the requirements of AA broiler management and provided a 24-h light per day. Vaccinations and veterinary care are not performed.

### 4.2. Sample Collection

According to the average body weight, eight broilers were selected from each treatment, one for each replicate. The chicks were slaughtered via exsanguination by cutting the jugular vein after a 12 h fast at 21 d old. The duodenum, jejunum, and ileum segment (about 1 cm from the midpoint) were fixed in 4% paraformaldehyde solution for 24 h to analyze the morphology of the small intestine. Chyme samples from the jejunum and ileum were collected and frozen at −20 °C to measure short chain fatty acid (SCFA) concentration and digestive enzyme activity. Additionally, some ileal chyme samples were stored at −80 °C for 16S rRNA gene sequences analyses. Then, the jejunum and ileum were opened, and intestinal mucosa samples were collected and stored at −20 °C to determine antioxidant capacity.

### 4.3. Growth Performance Measurement

The body weight and feed consumption for each replicate were recorded on days 1, 21 and 42. Body weight was measured after fasting for 8 h. The growth performance is expressed by body weight gain (BWG) and feed intake (FI) during each period (days 1–21, 22–42, and 1–42). Moreover, the ratio of feed intake to body weight gain (F/G) during each period (days 1–21, 22–42, and 1–42) were calculated out to evaluate the feed conversion ratio (FCR).

### 4.4. Intestinal Histomorphology Analyses

Paraformaldehyde-fixed intestine samples were dehydrated, and then embedded in paraffin. The 5 μm consecutive sections of each sample were prepared for morphological observations. After the sections were stained with hematoxylin eosin (H&E), images were captured using a DM300 microscope (Leica Microsystems, Wetzlar, Germany). Villus length (VH) and crypt depth (CD) were measured, and then the ratio of VH to CD (VH/CD) was calculated out [26].

### 4.5. Digestive Enzyme Activity Examination 

Chyme samples of the jejunum and ileum (about 0.5 g) were added with 9 times the volume of cold normal saline and homogenized, and then centrifuged at 2500 rpm for 10 min at 4 °C to form 10% homogenized supernatant. The activities of amylase, lipase and trypsin were determined using commercial kit (Nanjing Jiancheng Bioengineering Institute, Nanjing, China). The final activities of the above enzymes were standardized by total protein (TP) in the homogenized supernatant. The content of TP was measured using Pierce^®^ BCA protein assay kit (Thermo Scientific Inc., Waltham, MA, USA). 

### 4.6. Antioxidant Indices Examination

Homogenized supernatant of mucosa samples from jejunum and ileum were prepared as described above. Total antioxidant capacity (TAC), superoxide dismutase (SOD) and malondialdehyde (MDA) were measured using commercial kits (Nanjing Jiancheng Bioengineering Institute, Nanjing, China). The final results of the above indices were standardized by TP in the homogenized supernatant. The content of TP was measured using the Pierce^®^ BCA protein assay kit (Thermo Scientific Inc., Waltham, MA, USA). Moreover, the ratio of TAC to MDA (TAC/MDA) was calculated to evaluate the antioxidant/oxidative balance of mucosa. 

### 4.7. Short-Chain Fatty Acids (SCFA) Concentration Determination

Chyme samples (1.5 g) of duodenum jejunum and ileum were placed into 10-mL centrifuge tubes and mixed fully with 5.0 mL ddH_2_O to form a mixture. After overnight at 4 °C, the above mixture was centrifuged at 10,000 rpm for 10 min at 4 °C to collect supernatant, and the sediment was washed twice with ddH_2_O. Finally, the supernatant was volumetric to 10.0 mL. The concentrations of SCFA including acetic acid (AA), propionic acid (PA), and BA were determined using Agilent 6890 gas chromatography (Agilent Tecnologies, Inc., Palo Alto, CA, USA) as described previously [47]. 

### 4.8. Bacterial 16S rRNA Gene Analyses

Microbial community genomic DNA extraction, 16S rRNA gene amplification and sequencing of ileal chyme samples from six replicates of each treatment were carried out, as described previously [43]. In brief, microbial community genomic DNA was extracted using E.Z.N.A.^®^ soil DNA Kit. The V3–V4 hypervariable region of 16S rRNA gene from bacteria was amplified using a specific primer (338F, 5′-ACTCCTACGGGAGGCAGCAG-3′; 806R, 5′-GGACTACHVGGGTWTCTAAT-3′) using an ABI GeneAmp^®^ 9700 PCR thermocycler (Applied Biosystems Inc., CA, USA). Amplicons were sequenced using Illumina MiSeq platform (Illumina, San Diego, CA, USA). The raw reads were deposited into the NCBI Sequence Read Archive database (Accession Number: PRJNA782462). 

The data were analyzed using Majorbio I-Sanger Cloud Platform (https://cloud.majorbio.com/, accessed on 9 December 2021, Shanghai Majorbio Bio-Pharm Technology Co., Ltd., Shanghai, China). Operational taxonomic units (OTUs) with 97% similarity cutoff were clustered using UPARSE version 7.1 and removed chimeric sequences. Alpha diversity was evaluated by computing Sobs ACE, Chao 1, Shannon, and Simpson indices, and Beta diversity was analyzed by calculating the unweighted Unifrac distance and visualized using principal component analysis (PCoA).

### 4.9. Statistical Analyses

Statistical analyses of growth performance, intestinal morphology, digestive enzyme activity, antioxidant indices and SCFA concentration were performed using the SAS software version 9.4 (SAS Institute, Cary, NC, USA). Data were tested for significance by one-way ANOVA using the General Linear Models (GLM) procedure, following by Tukey’s multiple comparison tests, with the replicates as the experimental unit. Data are expressed as the mean ± SE. A *p*-value less than 0.05 was considered significant, and 0.05 < *p* < 0.10 was considered as a tendency towards significance.

## 5. Conclusions

In summary, the current study revealed that dietary 1000 mg/kg CSB supplementation improved the growth performance and the small intestinal development and function in 21 d old broilers, as evidenced by the enhancing VH/CD, increasing activities of digestive enzymes, antioxidant capacity, and SCFA concentrations. More important, the improved small intestinal early development is connected to the increased ileal microbial community diversity as well as the rise of *Akkermansia*. Consequently, the CSB is one of effective substitutes for in-feed antibiotics in the broiler industry. 

## Figures and Tables

**Table 1:** Effects of CBS on growth performance of broilers.

Item	CON	ANT	CSB
BWG, g			
D 1–21	552 ± 29.4 ^b^	573 ± 13.1 ^ab^	589 ± 42.8 ^a^
D 22–42	1349 ± 111 ^b^	1468 ± 88.1 ^a^	1428 ± 119 ^ab^
D 1–42	1902 ± 131 ^b^	2041 ± 84.5 ^a^	2017 ± 145 ^ab^
FI, g			
D 1–21	713 ± 19.5	700 ± 19.4	719 ± 40.2
D 22–42	2177 ± 125	2214 ± 105	2215 ± 186
D 1–42	2890 ± 142	2914 ± 103	2933 ± 215
F/G			
D 1–21	1.29 ± 0.0501 ^a^	1.22 ± 0.0256 ^b^	1.22 ± 0.0646 ^b^
D 22–42	1.62 ± 0.0861 ^a^	1.51 ± 0.0503 ^b^	1.55 ± 0.0644 ^ab^
D 1–42	1.52 ± 0.0615 ^a^	1.43 ± 0.0383 ^b^	1.46 ± 0.0436 ^b^

Abbreviations: D, day; BWG, body weight gain; FI, feed intake; F/G (FI/BWG), the ratio of feed intake to body weight gain; CON, the control diet; ANT, CON diet supplemented with antibiotics (enramycin, 8 mg/kg and aureomycin, 100 mg/kg); CSB, CON diet supplemented with 1000 mg/kg CSB. Values are expressed as the mean ± SE, n = 8; ^a,b^ mean values within a row with unlike superscript letters were significantly different (*p* < 0.05).

**Table 2:** Ingredient composition and nutrient levels of the basal corn–soybean meal diets.

Item (% Unless Noted)	1 to 21 Days Old	22 to 42 Days of Old
Ingredients		
Corn (7.9%, crude protein)	55.00	58.80
Soybean meal (43.6%, crude protein)	36.30	32.27
Soybean oil	4.15	5.00
Dicalcium phosphate	1.80	1.62
Sodium chloride	0.30	0.30
Limestone	0.90	0.67
Choline chloride (50%)	0.10	0.10
L-Lysine·HCl (99%)	0.21	0.10
DL-Methionine (98%)	0.24	0.14
Premix ^1^ (1%)	1.00	1.00
Total	100.00	100.00
Calculated Nutrient levels		
Metabolizable Energy (Mcal/kg)	2.97	3.06
Crude Protein	21.1	19.6
Available Phosphorus	0.46	0.39
Calcium	1.05	0.82
Lysine	1.32	1.10
Methionine	0.58	0.46

^1^ The premix provided the following per kg of diets: VA, 12,000 IU; VB_1_, 3.5 mg; VB_2_, 8.6 mg; VB_12_, 0.02 mg; VD_3_, 25,000 IU; VE, 20 IU; VK_3_, 32.5 mg; biotin, 0.20 mg; folic acid, 1.00 mg; D-pantothenic acid, 15 mg; nicotinic acid, 50 mg; Cu, 8 mg; Fe, 80 mg; Mn 120 mg; Zn 110 mg; Se 0.30 mg.

## Data Availability

The data presented in this study are available on request from the corresponding author. The data are not publicly available due to privacy.
